# Prevalence of precancerous lesions and other cervical abnormalities among internally displaced women in Benue State Nigeria

**DOI:** 10.11604/pamj.2024.47.50.39721

**Published:** 2024-02-07

**Authors:** Atenchong Ngwibete, Olayinka Ogunbode, Laadi Terrumun Swende, Mangalu Mobhe Agbada, Akinyinka Omigbodun

**Affiliations:** 1 Pan African University Life and Earth Sciences Institute (including Health and Agriculture)-PAULESI, University of Ibadan, Ibadan, Nigeria,; 2 Department of Obstetrics and Gynaecology, College of Medicine, University of Ibadan, Ibadan, Nigeria,; 3 Department of Family Medicine, Federal Medical Center Makurdi, Benue State, Nigeria,; 4 Département des Sciences de la Population et du Développement, Faculté des Sciences Economiques et de Gestion, Université de Kinshasa, Kinshasa, République Démocratique du Congo

**Keywords:** Internally displaced persons, precancerous lesions, Visual inspection with acetic acid (VIA), cervical cancer, Africa

## Abstract

**Introduction:**

visual inspection is a low-cost screening strategy that can be used to prevent cervical cancer in women. These techniques can improve screening health outcomes for internally displaced women (IDW) who have poor sexual and reproductive health and rights' behaviors and outcomes. This study aimed to determine the prevalence of precancerous lesions and other clinical features using a visual inspection with acetic acid (VIA) technique during a cervical cancer screening campaign in two internally displaced people (IDP) camps in Benue State, Nigeria.

**Methods:**

this was a cross-sectional study of 166 IDW who voluntarily participated in the study during a VIA cervical cancer screening campaign in two IDP camps in Benue State, Nigeria the screening was done by a group of qualified and trained healthcare workers and data was collected using a structured, pretested questionnaire.

**Results:**

a total of 99(60%) of the women had a first sexual experience at 16 years, while 78(47%) had more than 5 full-term pregnancies. Although only 72(43.4%) of the women acknowledged having more than one sexual partner, over 70% of the women stated that their sexual partner had another sexual partner. The prevalence of precancerous lesions among women was 10.8%. Smoking(p=0.003), age at menarche (p≤ 0.001) and sexual behaviors (p=0.009, p=0.004) were factors that had a statistically significant relationship with the presence of a precancerous lesion among the IDW. The study also highlights the high rate (95%) of cervicitis among the women and the relatively high rate (5.4%) of leukoplakia.

**Conclusion:**

the majority of IDW had sociodemographic and lifestyle characteristics that predisposed them to developing cervical cancer More targeted interventions aimed at improving the sociodemographic and lifestyle characteristics of IDW are recommended. In addition, there is a need to create awareness about cervical cancer among IDW and make screening available in camp facilities for early detection and management.

## Introduction

In Africa, cervical cancer is one of the most prevalent cancers among women of reproductive age (15-49 years) [1]. This cancer is caused by two types of human papillomavirus (HPV) strands (16 and 18) that are responsible for over 70% of precancerous lesions [2]. According to the WHO, over 111,632 new cases of cervical cancer were reported in sub-Saharan Africa in 2018. In the same year, cervical cancer was responsible for the deaths of 68% of women [3]. Cervical cancer is preventable and treatable if detected at an early stage, during the pre-cancerous stages. Hence, routine screenings of the cervix with simple and cost-effective procedures like the Visual Inspection with Acetic Acid (VIA) have been recommended for identifying pre-cancerous changes in the cells of the cervix, which can then be treated before they develop into cancer [4]. Visual inspection is able to detect a variety of abnormalities in addition to precancerous lesions and cervical cancer. These abnormalities include cervical inflammation, polyps, and sexually transmitted infections such as genital warts caused by HPV. Although these conditions may not always be precursors to cancer, they still have the potential to have significant clinical implications and may necessitate treatment.

Cervical cancer progresses slowly from the pre-cancer stage to invasive cancer. Even though the cost of treatment when cancer is detected at its precancerous stage is very low, poor access to preventive screenings and treatment services makes early detection a significant problem [5]. This has been linked to more than 90% of cervical cancer-related deaths [6]. Many factors have been identified as risk factors for cervical cancer. According to the WHO, the risk is higher in women who are: HIV-positive; have had multiple sexual partners; have sex with partners who have had multiple sexual partners; participate in high-risk sexual activity; have not been vaccinated against HPV; have a coinfection involving other sexually transmitted diseases; smoke; engage in long-term use of oral contraceptives; and have not been screened for precancerous lesions [3]. These risk factors are prevalent among displaced persons and puts them at risk of reproductive health issues including cervical cancers [7,8]. However, these women have poor access to reproductive health services and cervical cancer screening services. After a community health intervention to get more IDW in camps in Benue State to use sexual and reproductive health (SRH) services, a researcher found that not enough people knew about or used cervical cancer screening services. Following the health education intervention, women in the camps showed interest in taking up cervical cancer screening. Given that this service is unavailable at camp facilities, a free cervical cancer screening program was offered to the women. Data was collected during the screening services to assess the prevalence of precancerous lesions and associated clinical features among the IDW in two displaced camps in Benue State.

## Methods

**Study design/procedure:** the study was a cross-sectional study undertaken during a cervical screening exercise in an IDP camp. Following a health education intervention to improve the uptake of RH services (HIV/AIDS, family planning, and cervical cancer screening) in Daudu Camps 1 and 2, women showed interest in using cervical cancer screening services. However, this service was unavailable in the camp at the time of the intervention. Based on this, the researcher partnered with other healthcare workers to provide free cervical cancer screenings to the women in these camps.

**Study participants:** the screening program targeted displaced women between the ages of 18 and 50. Eligibility criteria included being within the age group, being sexually active, having an intact cervix, and not being pregnant. Participation in the screening program was not limited to persons who received the health education.

**Study location:** the study took place in Daudu, in the Guma local government area (LGA) of Benue State. This community hosts two IDP camps (Daudu Camp 1 and Camp 3). Most of the people living in these camps are indigenous people who had to leave their homes because of violence caused by land disputes between Fulani herders and farmers. Violence caused by land disputes between Fulani herders and farmers. Benue State is said to have over 1.5 million displaced persons, some of whom are in one of the 27 IDP camps [9].

**Screening procedure:** the screening was carried out by a team of six (6) healthcare professionals and four community healthcare workers. Each woman was given prescreening counseling before the procedure. During this session, data was collected with the consent of the participants. Each screening room had two health professionals. Upon insertion of a sterile Cusco's virginal speculum, 5% acetic acid was applied to the cervix using a spray bottle. Results were read after one minute of application of acetic acid with the aid of a bright touch light. Well-defined, visible acetowhite legions seen in the transformation zone were considered positive. For quality assurance, this was confirmed by a second trained healthcare provider. Acetowhite lesions on the cervix were confirmed using Lugols Iodine. Lesions smaller than 75% were treated with thermal ablation. The team also looked out for other features such as cervical polyps, cervicitis, condyloma or warts, and ectropion or erosion. The team treated people with infections, including cervicitis and precancerous lesion smaller than 75% and referred women with other clinical conditions for specialized care. Following the procedure, appropriate postoperative counseling was provided. All results were also recorded.

**Quality assurance:** the study included a team of six health care workers, including two doctors and four nurses, who were trained and experienced in performing cervical cancer screening using the visual inspection under acetic acid (VIA) and visual inspection with lugols iodine (VILI) techniques. These practitioners also had training in the use of the thermal ablator. Each screening room was made up of two healthcare professionals. A second healthcare provider confirmed the screening findings. To ensure that data was collected appropriately, the data collectors were trained in the use of the tool, which was created following a review of the literature with modifications made to suit the study objectives. The instrument was pretested on a group of IDW in another local government area within the State. The data collected used a face-to-face interview approach after verbal consent from the IDW. Just before each woman entered the screening room, she was interviewed for data collection. Women were also given Unique IDs to race data collected. After the screening, the women were given a result sheet which had their unique IDs, this was used to enter the results against the woman previously collected information.

**Variables of measure:** data collected during face-to-face interview included sociodemographic characteristics, life-style characteristics, and clinical features. These variables were selected following literature review on the subject. Sociodemographic characteristics included participants' age, marital status, educational level, income and age at menarche. Lifestyle characteristics included cigarette smoking, parity, age of first sex, age of first pregnancy, and the total number of pregnancies ever had. Clinical features assessed and recorded in the result sheet included, the presence of precancerous lesions, cervical polyps, cervicitis, condyloma or warts, and ectropion or erosion.

**Method data collection:** data collection was done with the help of the Kobo collect data collecting software. Four research assistants trained in use of the application collected the data. Each woman was given a unique Identification number which was used as an identifier during data collection.

**Method of data analysis:** the collected data was entered into SPSS version 23 for data analysis. Results were represented in frequency tables and simple bar charts. Bivariate analysis was done using the chi-squares test of independence to check for association between the presence of precancerous lesions and respondents characteristics.

**Ethical consideration:** the research emanated as a result of a quasi-experimental PhD intervention carried out to improve the knowledge and use of HIV, family planning, and cervical cancer screening services. Ethical approval for this intervention was obtained from the UI/UCH ethical approval committee. Further approval to conduct the screening activity and research was obtained from the state ministry of health MOH/STA/204/VOL.1/209.

## Results

**Sociodemographic characteristics of the study participants:** as seen in Table 1, the mean age of the women was 31 years ± 9.1 and up to 144 (86.7%) were married or had a live-in partner. A total of 111 (66.9%) had no formal education. Although 137 (82.5%) of the respondents were employed, 138 (83.1%) earned less than 18,000 Naira per month. Up to 95(57%) of women have their first menses at 15 years of age or later.

**Table 1:** sociodemographic characteristics of the study participants

Variables		Frequency (N)	Percentage (%)	Mean	Standard Deviation
Age in years				31.1	9.1
Age group (in years)	< 20	28	16.9		
	21-30	63	38.0		
	31-40	54	32.5		
	> 40	21	12.7		
Marital status	Married/have a live-in partner	144	86.7		
	Separated. /Divorced	7	4.2		
	Widowed	12	7.2		
	Single	3	1.9		
Highest level of Educational	None	111	66.9		
	Primary	19	11.4		
	Secondary	32	19.3		
	Tertiary	4	2.4		
Nature of employment	Unemployed	29	17.5		
	Employed	137	82.5		
Average monthly income (Naira)	< 18,000	138	83.1		
	18,000-99,000	25	15.1		
	>100,000	3	1.8		
Age at Menarche	< 14	95	57.2	14.1	2.1
	> 15	71	42.8		

**Lifestyle and behavioral factors of study participants**: Table 2 shows that smoking was prevalent among 6(3.6%) of the women. The mean age of first pregnancy was 16.7 years ± 4.4. Most of the women 78(47%) had more than 5 full-term pregnancies with a mean parity of 5±3 pregnancy. The mean age of sexual debut was 16 years ± 2 years of age with a majority 99(60%) having a first sexual experience at 16 years and below. A total of 72(43.4%) of the women had more than one sexual partner with more than three-quarter (72.9%) acknowledging having had a partner who had another sexual partner.

**Table 2:** lifestyle and behavioral factors of study participants

Variables		Frequency (N)	Percentage (%)	Mean	Standard Deviation
Cigarette smoking	No	160	96.4		
	Yes	6	3.6		
Number of sexual partners	1	74	56.6		
	> 1	72	43.4		
Ever had a sex partner who had another partner	No	45	27.1		
	Yes	121	72.9		
Age at first pregnancy	< 17	81	48.8	16.7	4.4
	> 18	85	51.2		
Parity	< 2	48	28.9	5	3
	3-4	40	24.1		
	> 5	78	47.0		
Sexual Debut	< 16	99	60.0		
	> 17	67	40.0	16	2

**Figure 1:**
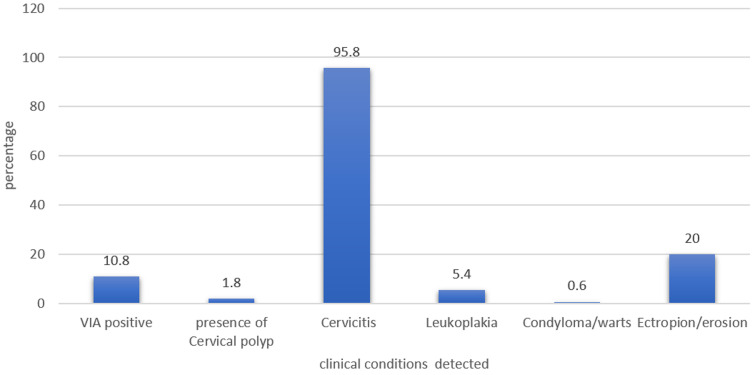
prevalence of clinical condition detected upon visual inspection

**Prevalence of clinical condition detected upon visual inspection:** most of the respondents had more than one of the abnormal clinical conditions during the inspection. As demonstrated in Figure 1, the most prevalent clinical condition detected during screening was cervicitis, with a prevalence of 95.9% and cervical ectropion with a prevalence of 20%. Leukoplakia was prevalent in 5.4% of the women. A total of 18 women (10.8%) had precancerous lesions (VIA positive) of which 14(8.4%) occupied less than 75% of the ectocervix.

**Association between precancerous lesions with risk factors:** according to the Table 3, smoking, age at menarche, having more than one sexual partner, age of first sex and having a sexual partner who had another sexual partner were factors that were significantly associated with the presence of a precancerous lesion among the IDW. Up to 5 of the 6 women had a precancerous lesion where smokers, contributing to 27.8% of those who were VIA-positive (χ^2^=33.8, p=< 0.001). Those who had more than one sexual partner contributed for up to 7.8% of the positivity (χ^2^=6.8, p= <0.001), while 10.8% of the positivity were of those who had sexual relationships with partners who had other partners (χ^2^=7.508, p=0.004). Women who had their first menarche at 14 and bellow had a high prevalence of a precancerous lesion (7.2%) this was statistically significant at χ^2^=4.7, P=0.003.

**Table 3:** bivariate association of precancerous lesions with risk factors

Variables		VIA Test Result					
		Negative		Positive		χ^2^	P value
		**n**	**%**	**n**	**%**		
History of STI	No	7	4.2	0	0.0	0.9**	0.346
	Yes	141	84.9	18	10.8		
Age in years	< 20	28	16.9	0	0.0	6.3	0.097
	21-30	55	33.1	8	4.8		
	31-40	45	27.1	9	5.4		
	> 40	20	12.0	1	0.6		
Marital status	Not living with a partner	20	12.0	0	0.0	2.76**	0.133
	Married or living with a partner	128	77.1	18	10.8		
Age of menarche	< 14	59	35.5	12	7.2	4.7	0.003*
	> 15	89	53.6	6	3.6		
Cigarette smoking	Never	147	88.6	13	7.8	33.8	<0.001*
	Former smoker	1	0.6	5	3.0		
Sexual debut	< 16	85	51.2	14	8.4	2.7**	0.070
	> 17	63	38.0	4	2.4		
Number of sex partners	1	89	53.6	5	3.0	6.8	0.009*
	>1	59	35.5	13	7.8		
Ever had a sex partner who had another partner	No	45	27.1	0	0.0	7.508**	**0.004**
	Yes	103	62.0	18	10.8		
Age at first pregnancy	< 17	71	42.8	10	6.0	5.4	0.622
	> 18	77	46.4	8	4.8		
Parity	< 2	46	27.7	2	1.2	3.2	0.20
	3 - 4	35	21.1	5	3.0		
	> 5	67	40.4	11	6.6		

*Test statistics is significant at 0.05 p value; ** fishers exact test.

## Discussion

The sociodemographic and lifestyle characteristics of the study population confirm that the majority of women in IDP camps have a live-in partner, low educational attainments, a low level of income, a high parity rate, a late age of menarche, and an early sexual debut. The women had a mean age of menarche of 14 years (2.1), with over 50% having their first menstrual flow at about 15 years or later. In Nigeria, the age of menarche has been reported to range between 12 and 15 years [10,11]. However, studies show that women in low-resource and rural settings who are likely to get poor anthropological measures begin menstruating at a mean age of 14 and above [10]. This may be the same reason for the relatively late menarche age among the women. Furthermore, the majority of the women lived below the poverty line, with a monthly income of less than 18000 Naira. This is indicative of a likelihood of poor living conditions and nutritional capability that may influence individual development and lifestyle choices. The fact that over 40% had more than one sexual partner, of which 72% had partners with another partner, confirms the influence of the women's low income on their lifestyle choices, which indirectly affect their SRHR.

Precancerous lesions were found in 10.8% of the respondents. Although there is a gap in research on the prevalence of precancerous cervical lesions among IDW, studies in Nigeria have found a prevalence rate ranging from 6 to 12% [12,13]. However, the prevalence of 12% was recorded among HIV-positive women, who are known to be at increased risk of developing cervical cancer [13]. Cervicitis was also a condition that was of high prevalence in the population (95.9%). Cervicitis is a cervix infection that manifests as an inflamed cervix with unusual discharge. Studies among the displaced population have proven that there is a high prevalence of reproductive tract infections among displaced women [14,15]. This can be attributed to the cultural, unhealthy sexual and reproductive health and poor hygienic practices among these women. Some of these practices include rape, unprotected sex, having more than one sex partner, unavailability of water and toilet facilities and poor housing conditions [16]. Some of these attributes were seen to be predominant among the women in this study; for example, up to 42% of the women acknowledged having had more than one sexual partner, with more than 70% acknowledging having had a sexual partner who had other partners. Literature has proven that these practices are not uncommon among women in displaced communities due to poverty, hardship, and poor knowledge about sexual health [17,18]. These same reasons may account for the high prevalence of cervicitis in the population.

Leukoplakia was noticed in 5.4% of women. According to Basu and Sankaranarayanan's publication on the WHO's International Agency for Research on Cancer (IARC) website, leukoplakia is a condition that causes white patches visible on the cervical epithelium even before acetic acid application. This patch comes as a result of keratin on the epithelium. Its etiology has been linked to HPV infection or to being idiopathic [19]. This prevalence is relatively high compared to that in the literature, which stated that leukoplakia is seen in 1 in 70 women [20]. This is of primary concern as studies have shown that 27% of cervical leucoplakia cases usually end up as cancer [20].

Bivariate analysis showed that smoking, age at menarche, having more than one sexual partner, and having a sexual partner who had another sexual partner were factors that had a significant associated with the presence of a precancerous lesion in the women. These same factors have been identified in other literature [13,21]. Other factors that have been found to significantly affect the presence of a precancerous lesion in a woman include a history of STIs, age at first sex, parity greater than five, and HIV status [1]. However, these factors were not significant in our study. This may be due to the fact that these characteristics were fairly common among the women in the study. According to the study, over 40% of the IDW had a parity of more than 5, over 90% had a history of STIs, and 60% had sexual intercourse at age 16 or below. Other studies in Nigeria and Africa have found a much lower prevalence of such traits [13,22]. Although there was no significant relationship between women's age and the presence of a precancerous lesion, the majority of the women with a lesion were between 21 and 30 years old (4.8%) and 30 and 40 years old (5.4%), respectively. No lesion was found in women 20 and below. On the contrary, many studies have found a significant relationship between a woman's age and the presence of precancerous lesions [12,23]. However, just like many studies, cervical cancer lesions have been found to exist more among women over 30 and less than 40 [24]. The study also confirms that precancerous lesions are less likely in women under 20 years of age.

## Conclusion

The research conducted substantiates the high occurrence of sociodemographic and lifestyle attributes among Internally Displaced Women (IDW) that make them susceptible to precancerous lesions. This investigation contributes to the existing literature by providing further evidence on the prevalence of precancerous lesions and other associated factors among IDW. In addition to the relatively high incidence of precancerous lesions, the study underscores the prevalence of related clinical conditions and lifestyle factors that suggest suboptimal sexual and reproductive health practices among this population. It highlights factors such as smoking, multiple sexual partners, early age of menarche, and having a sexual partner who is non-monogamous to significantly influence the occurrence of precancerous lesions among these women. Given the sociodemographic and lifestyle attributes of the IDW, it is imperative to raise awareness about cervical cancer within this group and ensure the availability of screening services in camp facilities for early detection and intervention. The study does have certain limitations. The sample was selected for convenience from the population, and data collection was restricted to only two IDP camps out of the 14 present in the study area, thereby limiting the number of women who could participate in the screening process. Despite these limitations, the study's strength lies in the expertise of the screening team.

### What is known about this topic

- *Internally displaced women have poor sexual and reproductive healthcare practices and access to sexual and reproductive rights services are poor;*
- *VIA technology is a cost-effective procedure that could be used for screening of cervical cancer in resource-limited and humanitarian settings*.

### What this study adds

- *IDW of the study population have high risk characteristics that predisposes them to cervical cancer;*
- *The prevalence of precancerous lesions among the IDW in the study population is high;*
- *Conditions such as cervicitis and leukoplakia also have a high prevalence in the study population*.
